# Castration-Resistant Prostate Cancer Refractory to Second-Generation Androgen Receptor Axis-Targeted Agents: Opportunities and Challenges

**DOI:** 10.3390/cancers10100345

**Published:** 2018-09-21

**Authors:** Yuki Kita, Takayuki Goto, Shusuke Akamatsu, Toshinari Yamasaki, Takahiro Inoue, Osamu Ogawa, Takashi Kobayashi

**Affiliations:** Department of Urology, Kyoto University Graduate School of Medicine, Kyoto 606-8507, Japan; kitayuki@kuhp.kyoto-u.ac.jp (Y.K.); goto@kuhp.kyoto-u.ac.jp (T.G.); akamats@kuhp.kyoto-u.ac.jp (S.A.); yamatosi@kuhp.kyoto-u.ac.jp (T.Y.); takahi@kuhp.kyoto-u.ac.jp (T.I.); selecao@kuhp.kyoto-u.ac.jp (T.K.)

**Keywords:** castration-resistant prostate cancer, heterogeneity, androgen receptor, drivers

## Abstract

Second-generation androgen receptor axis-targeted (ARAT) agents, namely abiraterone and enzalutamide, enable stronger blockade of the androgen receptor (AR) axis and longer survival of men with castration-resistant prostate cancer (CRPC). However, the extent of the improved survival remains insufficient and the majority of patients eventually develop resistance to these novel agents. Some patients develop resistance against ARAT treatment through mechanisms termed “complete AR independence” or “AR indifference”, and no longer require activation of the AR axis. However, a considerable proportion of CRPC patients remain persistently dependent on AR or its downstream signaling pathways. Ligand-independent activation of the AR, an AR axis-dependent mechanism, is mediated by truncated forms of ARs that lack the ligand-binding domain (LBD), arising as products of *AR* splicing variants or nonsense mutations of *AR*. Post-translational modifications of ARs can also contribute to ligand-independent transactivation of the AR. Other mechanisms for AR axis activation are mediated by pathways that bypass the AR. Recent studies revealed that the glucocorticoid receptor can upregulate a similar transcription program to that of the AR, thus bypassing the AR. ARAT agents are essentially ineffective for CRPC driven by these AR-independent mechanisms. This review article describes recent efforts to overcome these refractory machineries for the development of next-generation AR axis blockade in CRPC.

## 1. Introduction

In the United States, prostate cancer (PC) is the most commonly diagnosed malignancy, and the second highest cause of cancer-related deaths among men. It is estimated that 164,690 new cases of PC will be diagnosed and 29,430 patients will die of PC in 2018 [1]. PC is dependent on androgen receptor (AR) activity for its initiation and progression. The standard treatment for advanced PC is androgen-deprivation therapy (ADT), which suppresses the transcriptional activity of the AR. Most tumors initially shrink in response to ADT, but eventually become castration-resistant. In recent years, castration-resistant PC (CRPC) was shown to remain dependent on the AR signaling axis despite systemic depletion of androgens by various mechanisms [2]. Two novel agents, abiraterone and enzalutamide, were developed as second-generation AR axis-targeted (ARAT) agents that provide more potent inhibition of the AR pathway.

Abiraterone acetate is an oral drug that selectively and irreversibly inhibits the CYP17A1 microsomal enzyme, leading to inhibition of testosterone biosynthesis in the testes, adrenal glands, and PC cells. Enzalutamide is an oral AR antagonist with eight-fold higher affinity for its target than bicalutamide [3], and also prevents AR nuclear translocation and DNA binding. These drugs have been approved by the US Food and Drug Administration (FDA) for clinical use because they showed significant survival benefits in both docetaxel-resistant [4,5,6] and docetaxel-naïve [7,8] settings.

Despite the significant survival benefits for treatment groups compared with placebo groups, some individuals fail to respond to these drugs from the beginning of treatment (e.g., no initial prostate-specific antigen (PSA) response) because of intrinsic resistance. Although the remaining patients initially respond to treatment, they eventually develop acquired resistance, typically within a few months.

Treatment of ARAT-refractory CRPC is a clinical challenge, because effective treatment in this setting is quite limited. Docetaxel, cabazitaxel, sipuleucel-T, and Ra-223 are current candidates for patients with ARAT-refractory CRPC, but their survival benefits are very limited or remain unproven in a restricted sense. Moreover, treatment with ARAT agents can significantly alter the biological characteristics of CRPC, and consequently require specific attention in the management of ARAT-refractory CRPC.

This article reviews the current understanding of the molecular bases underlying intrinsic and acquired resistances to second-generation ARAT agents, focusing on their relationships with the AR signaling axis, and summarizes novel treatment strategies based on these mechanisms.

## 2. Mechanisms for Resistance to ARAT Agents Classified by AR Signaling Activation

**Figure 1:**
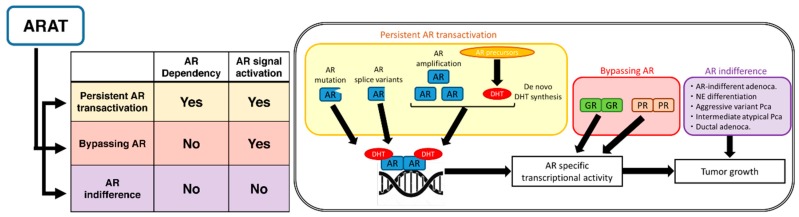
Suggested mechanisms of resistance to second-generation androgen receptor axis-targeted (ARAT) agents, classified into three groups by their dependency on the androgen receptor (AR) and its downstream signals: persistent AR transactivation, bypassing AR, and AR indifference. DHT = dihydrotestosterone; NE = neuroendocrine; Pca = prostate cancer.

Several reported mechanisms for resistance to second-generation ARAT agents can be classified into three groups by their dependency on AR and its downstream signals [9,10,11,12,13] (Figure 1).

### 2.1. Persistent AR Transactivation

In this category, despite castration levels of serum testosterone, some mechanisms still activate AR to induce expression of downstream target genes. Activation of the altered androgen biosynthesis pathway in a tumor, amplification and overexpression of the AR gene, genetic mutation of the AR, constitutively active AR splice variants, and phosphorylation of the AR by the Src and Akt pathways have been reported as possible mechanisms. These mechanisms can be further classified by the presence or absence of ligand dependency.

#### 2.1.1. Ligand-Dependent Activation of AR

##### De Novo Synthesis of DHT

Although abiraterone inhibits CYP17A1 and suppresses androgen synthesis, studies involving animal models have shown that CYP17A1 is overexpressed in tumors and that key genes involved in the androgen synthesis pathway, such as CYP11A1, AKR1C3, and HSD17B3, are upregulated during treatment [14,15]. Since abiraterone cannot completely ablate serum precursor steroids like dehydroepiandrosterone sulfate (DHEA-S), a mechanism by which these upregulated enzymes produce testosterone via the so-called classical pathway in tumor cells is conceivable. 

A gain-of-function N367T mutation in 3-beta-hydroxysteroid dehydrogenase type 1 (3βHSD1) generated during treatment with abiraterone can inhibit degradation of 3βHSD1 and promote DHT synthesis from DHEA via 5α-androstenedione as an alternative pathway [16]. Likewise, aldo-keto reductase 1 (AKR1C3), whose expression is upregulated in PC cells resistant to enzalutamide and abiraterone, can be responsible for resistance because it enables DHT biosynthesis through alternative pathways. Treatment of abiraterone-resistant cells with indomethacin, an AKR1C3 inhibitor, can overcome resistance and enhance abiraterone therapy both in vitro and in vivo by reducing the levels of intracrine androgens and diminishing AR transcriptional activity [17,18].

##### AR Amplification/Overexpression

In 1995, Taplin and colleagues reported that AR expression was elevated in more than 80% of CRPC cases [19]. Moreover, AR amplification, leading to AR overexpression, enables progression to CRPC even in the setting of low circulating androgens with ADT [20]. A similar phenomenon was even reported in the era of second-generation ARAT agents. Increased expression of the full-length AR and the truncated AR in PC cells after administration of abiraterone or enzalutamide was observed in in vitro and in vivo models [14,21]. Furthermore, several analyses of circulating tumor DNA (ctDNA) in patients showed that AR gene amplification was associated with resistance to treatment with second-generation ARAT agents [22,23,24,25,26]. However, it remains unknown how AR gene amplification contributes to enzalutamide resistance. Furthermore, because expression of AR-V7, an AR splice variant, is often associated with AR gene amplification, it remains controversial which of the two truly drives drug resistance.

##### AR Mutations

In CRPC, AR mutations are found in 5–30% of tumors, circulating tumor cells (CTCs), and ctDNA [22,24,27]. The majority of clinically-relevant somatic mutations in AR, including L702H, H875Y, F877L, and T878A, are located in the LBD.

AR F877L (previously reported in the literature as F876L), a phenylalanine-to-leucine substitution at amino acid 877, was shown to convert enzalutamide from a potent antagonist to a partial agonist in PC cell lines [28,29,30]. Spontaneous F877L mutations were detected in ctDNA of patients treated with enzalutamide [22].

AR T878A (previously reported as T877A) is a missense mutation that leads to AR activation by progesterone and first-generation antiandrogens such as flutamide. Since inhibition of CYP17A1 by abiraterone promotes elevated expression of intracellular progesterone in tumor tissues, while DHEA and testosterone are suppressed, it is suggested that the T878A mutation creates malignant clones to overcome abiraterone inhibition. Chen et al. [31] reported that T878A was detected in three of 18 CRPC tissues resistant to abiraterone.

L702H is a mutation that leads to glucocorticoid-mediated activation of ARs. Carreira et al. [32] revealed that the L702H mutation was associated with primary resistance to abiraterone.

To overcome resistance by inhibiting these mechanisms of ligand-dependent AR transactivation, several clinical trials on approaches such as combined administration of enzalutamide and abiraterone, dose escalation of abiraterone, novel AR antagonists, and progesterone receptor inhibitors are underway [33].

#### 2.1.2. Ligand-Independent Activation of AR

##### AR Splice Variants

In CRPC, the splicing mechanism for AR mRNA produces splice variants that lack the LBD, which plays an important role in cancer proliferation under androgen ablation [34,35]. Among the various splice variants lacking the LBD, AR-V7 has been most extensively investigated because it was identified in a CRPC cell line in early studies and was reported to be increased in CRPC clinical specimens [36]. In 2014, it was reported that expression of AR-V7 in CTCs could be a predictive biomarker for drug resistance against enzalutamide or abiraterone [37]. In the study, CTCs from 31% of enzalutamide-treated patients and 19% of abiraterone-treated patients exhibited detectable AR-V7 mRNA expression, and AR-V7-positive patients had significantly lower rates of PSA response and experienced significantly shorter progression-free survival and overall survival.

Regarding AR-V7 expression, one mechanism is that ADT-induced AR gene transcription and the recruitment of splicing factors like U2AF65 and alternative splicing factor/pre-mRNA-splicing factor SF2 (ASF/SF2) to AR pre-mRNA contribute to enhanced AR-V7 levels in PC cells [38]. Another mechanism is structural alterations in the AR gene [39,40]. Since the latter is a permanent mechanism that induces high levels of AR-V7 expression, it is speculated that AR gene abnormalities are a powerful cause of cell proliferation under ADT. In fact, analyses using clinical specimens and CTCs from CRPC patients that acquired resistance to enzalutamide or abiraterone revealed structural abnormalities of AR in many cases, in which significantly increased expression of various AR splice variants lacking the LBD was also observed [41,42]. 

Many studies have focused on the functional significance of AR-V7. AR-V7 retains the nuclear localization signal and promotes the expression of target genes by translocation in a ligand-independent manner [43]. Therefore, growth suppression does not occur, even under administration of novel AR antagonists like enzalutamide [44]. AR-V7 can also form dimers with wild-type ARs and other AR variants [45]. However, it remains controversial whether AR-V7 function requires the presence of the wild-type AR [46].

Additionally, in-depth studies have also been conducted on other AR variants, including AR-V1, AR-V3, AR-V9, and ARv567es [47,48,49]. Since these variants also contain the AR DNA-binding domain and the AR transcriptional activation domain, they are capable of transcriptional regulation, in spite of the loss of the AR-LBD. Therefore, they are not regulated by either first-line or novel hormonal therapies. However, whether AR variants drive therapeutic resistance in CRPC remains an unresolved topic. Nyquist et al. showed the model where AR variants were endogenously expressed at high levels, and their knockdown restores sensitivity to castration and anti-androgens [50]. In contrast, some studies suggested that rapid induction of AR variants by ADTs might be a by-product of the increased transcription of the AR gene and simply reflects a mechanism for rapid induction of full length AR expression by ADTs [51,52]. Further studies are warranted to elucidate the role of AR variants as novel factors mediating resistance to CRPC.

##### AR Mutant

Han et al. [53] identified the novel AR mutant Q874 in biopsy tissues from patients who had acquired resistance to a CYP17 inhibitor. The mutant protein has no transcriptional activity by itself, similar to other AR splice variants, but forms a heterodimer with the full-length AR to enhance its transcriptional activity.

##### Crosstalk with Other Oncogenic Signaling Pathways

In the presence of low androgen levels, the AR interacts with Src kinase and the p85α regulatory subunit of phosphoinositide 3 kinase (PI3K) to activate mitogen-activated protein kinase (MAPK) and Akt pathways and enhance cell proliferation and survival in a non-genomic manner. Activated MAPK and Akt by non-genomic signaling also enhance genomic AR signals by phosphorylating the AR or transcriptional coactivators [54,55]. Thomas et al. [56] showed that synergistic targeting of the PI3K/Akt pathway and the AR axis significantly delayed CRPC progression in in vivo models. Many such strategies are currently under investigation and have shown promising results in preclinical models [57,58,59,60].

To establish novel treatments that can suppress non-genomic AR signaling, several clinical trials of combination therapy with second-generation ARAT agents and molecular targeted drugs such as tyrosine kinase inhibitors are currently being conducted.

### 2.2. Bypassing AR

This category means that AR downstream genes are expressed by activation of not the AR, but other receptors or pathways. Mechanisms involving the glucocorticoid receptor (GR) and the progesterone receptor (PR) have been reported as described below.

#### 2.2.1. GR Overexpression

Resistance to enzalutamide or abiraterone can occur through increased expression of the GR because it shares response elements with the AR in multiple gene targets. Arora et al. [61] reported that GR overexpression induced expression of several AR target genes independently of the AR to confer clinical resistance against enzalutamide. For this reason, the GR agonist dexamethasone induces enzalutamide resistance, while a GR antagonist restores enzalutamide sensitivity in vitro.

These findings suggest that combined inhibition of GRs and ARs could be a promising treatment strategy. Kach et al. [62] reported that two novel non-steroidal and highly selective GR modulators, CORT118335 and CORT108297, can block GR activity in PC and slow CRPC progression in vivo, thereby demonstrating the therapeutic potential for GR-expressing CRPC.

#### 2.2.2. PR Upregulation

PR expression is increased in CRPC, and two isoforms (A and B) are expressed in interstitial fibroblasts and smooth muscle cells in the prostate to regulate cell proliferation. Sustained production of progesterone by enzalutamide or abiraterone may be involved in acquired resistance [63].

### 2.3. AR Indifference

In this category, tumor cells proliferate under ADT without activation of the AR or expression of AR target genes.

Neuroendocrine prostate cancer (NEPC) is a clinically aggressive subtype of PC with pathological features of neuroendocrine differentiation that are enriched in the advanced disease setting after ADT [64]. Although the mechanism for its occurrence remained a mystery for a long time, it has gradually become apparent in recent studies. Several groups found that AR-regulated *TMPRSS2-ERG* genomic translocation in AR-negative small-cell carcinoma also existed in AR-positive adenocarcinoma [65,66,67]. In addition, whole-exome sequencing revealed significant overlaps in the mutational and copy number landscapes between castration-resistant adenocarcinoma and NEPC, supporting clonal evolution of NEPC from adenocarcinoma [68]. Moreover, in PC preclinical models, the combination of RB1 and TP53 could drive the development of NEPC, suggesting that *RB1* and *TP53* can contribute to its pathogenesis [69,70]. These tumors exhibit resistance to enzalutamide through a phenotypic shift from AR-dependent luminal epithelial cells to AR-independent basal-like cells. Meanwhile, Zou et al. [71] showed that focal and overt neuroendocrine regions arise through transdifferentiation of luminal adenocarcinoma cells using lineage tracing analyses in a genetically engineered mouse model. Another study identified the involvement of a placental gene, PEG10, in NEPC transdifferentiation and invasion through transcriptional re-expression and translational stabilization of PEG10 via AR-, p53-, and RB1/E2F1-dependent mechanisms in patient-derived xenografts [72].

## 3. Next-Generation ARAT Agents

In recent years, development of novel drugs has been underway for efficient targeting of the above-described ligand-dependent and ligand-independent AR activation processes. These drugs include agents that antagonize the AR more strongly, degrade the AR, target the N-terminal and DNA-binding domain of the AR, or inhibit AR dimer formation [21,73,74,75,76] (Table 1).

Apalutamide (ARN-509) is a non-steroidal anti-androgen agent that binds directly to the LBD of the AR and prevents AR translocation, DNA binding, and AR-mediated transcription, similar to enzalutamide. Recently, the phase three SPARTAN trial showed that metastasis-free survival and time to symptomatic progression in men with non-metastatic CRPC were significantly prolonged by apalutamide compared with placebo [73]. It was originally considered that there was no clear clinical niche for apalutamide because of its overlap with enzalutamide in structure, mechanism, and pharmacology. However, accumulated data from these clinical trials suggest that apalutamide differs from enzalutamide in its adverse effect (AE) profiles: it is associated with much less fatigue and lower gastrointestinal toxicity, but more frequent skin rash. Management of drug-associated AEs would be important, particularly because these new-generation drugs are increasingly used for the early stage of CRPC and patients are expected to be on the drugs for a long duration.

Darolutamide (ODM-201) is an oral, investigational, high-affinity AR antagonist with activity against known AR mutants like F877L, W741L, and T878A that confer resistance to other second-generation anti-androgens, has minimal brain barrier penetration, and does not significantly increase serum testosterone [77]. A phase three clinical trial in patients with non-metastatic CRPC is currently underway (NCT02200614).

TRC253 is a novel high-affinity competitive inhibitor of the AR. TRC253 is also a pan-inhibitor of multiple AR mutants, including F877L, and is under development for treatment of men with PC in a phase one/two clinical study (NCT02987829).

Seviteronel (VT-464) is a non-steroidal anti-androgen that specifically acts as an androgen synthesis inhibitor through inhibition of the enzyme CYP17A1. It has approximately 10-fold selectivity for the inhibition of 17,20-lyase over 17α-hydroxylase, resulting in less interference with corticosteroid production relative to abiraterone acetate, and could thus be administrable without a concomitant exogenous glucocorticoid. In addition, seviteronel appears to exert greater efficacy as an anti-androgen than abiraterone in vitro [78]. Several phase two clinical trials are underway in CRPC patients previously treated with enzalutamide or abiraterone (NCT02130700, NCT02445976, NCT02012920).

Galeterone (TOK-001) is a selective multi-targeted agent that inhibits CYP17, antagonizes the AR, and reduces AR expression in PC cells by increasing AR degradation [79]. A phase one/two study (ARMOR1/2) showed that galeterone was well-tolerated and led to pharmacodynamic changes consistent with its selective multifunctional AR signaling inhibition [80]. Because its multifocal mechanisms may be effective for AR-V7, a randomized phase three trial (ARMOR3-SV) comparing galeterone and enzalutamide in treatment-naïve metastatic CRPC patients with AR-V7 positive CTCs was carried out, but failed to show a significant benefit (NCT02438007).

Niclosamide, an FDA-approved anti-helminthic drug, was identified as a potent AR-V7 inhibitor in PC cells in a screening of known compounds [74]. Niclosamide significantly downregulates AR-V7 protein expression by protein degradation through a proteasome-dependent pathway, inhibits AR-V7 transcription activity, and reduces AR-V7 recruitment to the PSA promoter. Niclosamide inhibits PC cell growth in vitro and tumor growth in vivo. Furthermore, the combination of niclosamide and enzalutamide results in significant inhibition of enzalutamide-resistant tumor growth, suggesting that niclosamide can enhance enzalutamide therapy and overcome enzalutamide resistance in CRPC cells. However, a phase one clinical trial of niclosamide in combination with enzalutamide showed disappointing results because oral niclosamide could not be escalated above sufficient doses, and its plasma concentrations were not consistently above the threshold shown to inhibit growth in CRPC models [81].

EPI-506 is a pro-drug for one of the four stereoisomers of its predecessor, EPI-001. EPI analogs covalently bind to the AR N-terminal domain to block the transcriptional activity of AR and its splice variants and reduce the growth of CRPC xenografts [76,82]. A phase one/two clinical trial of EPI-506 in patients with metastatic CRPC is currently underway (NCT02606123).

## 4. Future Perspectives

### 4.1. Molecular Mechanisms for AR Overexpression

As described above, AR amplification drives PC toward castration-resistant progression. However, it has been difficult to target AR amplification because the molecular mechanisms for AR amplification have been unknown. In this regard, Takeda et al. [83] recently reported that activation and amplification of an AR enhancer upstream of the AR locus drove the progression of metastatic CRPC. The AR enhancer is frequently amplified in CRPC clinical samples, and insertion of an additional copy of this region sufficed to increase proliferation under low androgen conditions and to decrease sensitivity to enzalutamide in vitro. A subsequent report of linked-read genome sequencing data from patients by Viswanathan et al. [84] revealed that the majority of cases had duplications of an AR enhancer that was associated with progression under androgen pathway inhibitors. The activated and amplified enhancer is likely to become a new therapeutic target for suppression of AR expression. Although targeting of transcription factors with small molecules has been a pharmacological challenge, emerging technologies such as pyrrole-imidazole polyamides, which are small synthetic molecules that recognize and attach to the minor groove of DNA with high affinity and sequence specificity [85], may overcome the problem.

### 4.2. How Can We Know the Real Driving Mechanism in Individual Patients?

As the mechanism of the disease has continued to become more apparent, the number of new therapeutic agents have increased. However, this has brought about new clinical challenges. First, it is important for doctors to predict which drugs can be effective, but research on predictive factors including biomarkers has not yet progressed. With the increasing importance of companion diagnosis, liquid biopsy has attracted much attention in PC, because tissue sampling from metastatic lesions is often difficult. Annala et al. [27] reported the relative impacts of common ctDNA alterations on patient responses to enzalutamide and abiraterone by leveraging plasma specimens collected in a randomized phase two trial. Their findings suggest the potential usefulness of liquid biopsy analysis for guiding the use of AR-targeted therapy in general practice.

### 4.3. Novel Therapeutic Agents Multiply Questions for Clinical Use

Given the fact that no medicine can be expected to provide a cure for CRPC, novel therapeutic agents multiply questions for clinical use, such as whether to use the agents in combination or in sequence, how to set goals for treatment with individual medications, how to judge their effectiveness, and how to decide whether to change treatment. Further studies are needed to resolve these questions, and clinical trials based on multi-arm multi-stage platform protocols such as the STAMPEDE trial [86] may become very important. Such protocols are suitable for the current situation, wherein treatment options are increasing, because comparisons of a standard-of-care (SOC) arm with multiple test arms can reduce the number of cases required and can be flexibly modified when new drugs emerge or when the SOC changes.

### 4.4. Heterogeneity Is Multi-Dimensional

**Figure 2:**
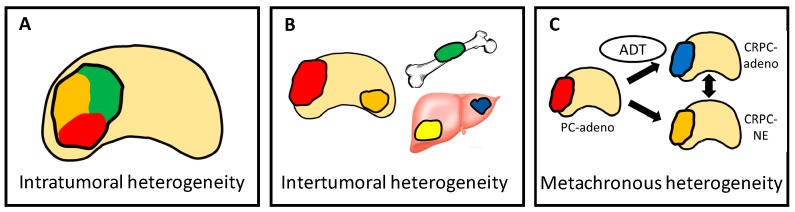
Multi-dimensional classes for tumor heterogeneity, including four types of diversity: (**A**) intra-tumoral, (**B**) inter-tumoral, and (**C**) metachronous heterogeneity. Tumors with different colors represent different clonalities or biological properties.

Despite the increasing number of treatments proven to prolong overall survival, none of them have shown promising results in terms of a cure. One of the main reasons for this appears to lie in the associated heterogeneity. There are multi-dimensional classes for tumor heterogeneity including four types of diversity (Figure 2). Tumor cells may develop intrinsic adaptations driven by multiple drivers during their progression in response to microenvironmental changes or therapeutic pressure. An adaptation pathway via reciprocal feedback activation of PI3K signaling upon blockade of AR signaling is a typical example [55]. At the same time, another dimension of divergence may evolve within growing tumor foci (intra-tumoral heterogeneity). When the disease has disseminated, diversity among the primary and metastatic lesions also exists (inter-tumoral heterogeneity). In addition, CRPC cells can modify themselves over time with treatment, as represented by lineage plasticity (metachronous heterogeneity). These heterogeneities are considered to form the basis of resistance.

### 4.5. Overcoming Heterogeneity that Drives Treatment Resistance

**Figure 3:**
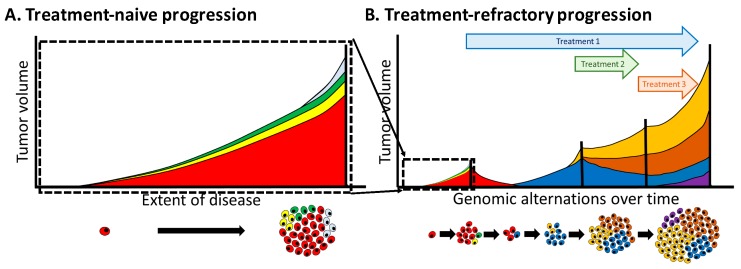
Schematic illustration depicting the extent of tumor heterogeneity. As the disease progresses in nature (**A**) or against therapeutic pressure (**B**), the extent of tumor heterogeneity is exponentially increased.

The pivotal mechanisms associated with treatment resistance of metastatic CRPC are clearly inter-tumoral and metachronous heterogeneities. It seems very difficult, at least to date, to treat such cases once the disease has acquired these high extents of heterogeneity and plasticity, although recent reports have shown promising results for reversing neuroendocrine differentiation of CRPC [69,70]. In this regard, it appears more efficient to invest in potent treatments at earlier stages before the extent of tumor heterogeneity can become exponentially increased according to disease progression. In the treatment-naïve state, intra-tumoral heterogeneity increases as the tumor burden increases, and individual metastatic lesions are affected by their local microenvironment to acquire further inter-tumoral heterogeneity, even though they are relatively homogeneous at the genetic level (Figure 3A). Furthermore, the metachronous heterogeneity increases based on the plasticity induced by ADT and subsequent treatment (Figure 3B). Therefore, a reasonable possibility to overcome such divergence-based resistance may exist in starting multiple and potent therapies targeting heterogeneous mechanisms, including adaptation pathways, at a relatively early stage when heterogeneity has not increased. Indeed, upfront docetaxel or abiraterone in combination with ADT has shown the usefulness of early multiple treatments [87,88,89,90].

Unfortunately, most of these studies targeted only high-risk cases or failed to prove the superiority of multiple therapies in low-risk cases. It remains unknown whether a subset of low-risk cases may be cured by multiple treatments more frequently compared with ADT alone. A substantial proportion of patients with low-risk PC achieve durable responses with almost complete remission by ADT alone, which lowers the benefit of combined treatment. Instead of the current prognostic risk stratification, precise prediction of treatment responses based on better understanding of potentially heterogeneous mechanisms will be essential for this strategy. Ideally, treatment drugs should be selected in a personalized manner, incorporating multiple factors of the disease and the patient (e.g., age, performance status, comorbidities). Companion diagnostics using genetic tests, liquid biopsy, and other technologies will be increasingly available for clinical decision-making for multiple treatments in the first-line early intervention setting. Otherwise, the number needed to treat and time needed for follow-up will be much larger to examine the benefit of combined treatment in this population.

Clearly, management of AEs accompanying multiple treatments is very important. Increased cost of the treatments is another inevitable issue. In summary, further studies are warranted to understand and evaluate tumor heterogeneity in CRPC. When we aim to cure patients with advanced PC, multiple personalized treatments based on the simultaneous use of potent weapons to the right patient at the right time in the right way with good control of associated AEs and using not only medicine but also surgery and radiotherapy are desirable.

## 5. Conclusions

The widespread use of second-generation ARAT agents has multiplied novel biological and clinical questions, including increased tumor heterogeneity. More effort is needed to clarify unmet clinical needs and underlying biological mechanisms, and to find a solution that can be applied to patients by frequently going back and forth between the benchside and the bedside.

## Figures and Tables

**Table 1:** Novel AR-targeted drugs in clinical trials.

Agents	Mechanism of Action	Clinical Trials
Apalutamide (ARN-509)	Second-generation AR antagonist	NCT01946204 (SPARTAN trial)
Darolutamide (ODM-201)	Second-generation AR antagonist	NCT02200614
TRC253	Second-generation AR antagonist	NCT02987829
Seviteronel (VT-464)	Lyase-selective inhibitor of CYP17A1	NCT02130700 NCT02445976 NCT02012920
Galeterone (TOK-001)	Dual CYP17 inhibitor and AR antagonist	NCT02438007
EPI-506	N-terminal domain AR inhibitor	NCT02606123

AR: androgen receptor.
